# Circularity of islets is a distinct marker for the pathological diagnosis of adult non-neoplastic hyperinsulinemic hypoglycemia using surgical specimens

**DOI:** 10.1186/s13000-023-01403-y

**Published:** 2023-10-20

**Authors:** Ryota Nakagawa, Sachiko Minamiguchi, Tatsuki R. Kataoka, Junji Fujikura, Toshihiko Masui, Masakazu Fujimoto, Yosuke Yamada, Yasuhide Takeuchi, Yuki Teramoto, Hiroaki Ito, Manduwa Saka, Kyohei Kitamura, Shinya Otsuki, Ryohei Nishijima, Hironori Haga

**Affiliations:** 1 Department of Diagnostic Pathology, Kyoto University Hospital, Sakyo-ku, Kyoto, 606-8507 Japan; 2 Department of Pathology, Iwate Medical University, Yahaba-cho, Shiwa-gun, Iwate, 028-3694 Japan; 3 Department of Diabetes, Endocrinology and Nutrition, Kyoto University Hospital, Sakyo-ku, Kyoto, 606-8507 Japan; 4 Department of Department of Hepatobiliary Pancreatic Surgery and Transplantation, Kyoto University Hospital, Sakyo-ku, Kyoto, 606-8507 Japan

**Keywords:** Adult non-neoplastic hyperinsulinemic hypoglycemia (ANHH), Circularity, Digital pathology, Image analysis, Islets, Nesidioblastosis, NIS-Elements, Quantitative criteria

## Abstract

**Background:**

Adult non-neoplastic hyperinsulinemic hypoglycemia (ANHH), also known as adult-onset nesidioblastosis, is a rare cause of endogenous hyperinsulinemic hypoglycemia in adults. This disease is characterized by diffuse hyperplasia of pancreatic endocrine cells and is diagnosed by a pathological examination. While diagnostic criteria for this disease have already been proposed, we established more quantitative criteria for evaluating islet morphology.

**Methods:**

We measured the number, maximum diameter, total area, and circularity (representing how closely islets resemble perfect spheres) of islets contained in representative sections of ANHH (n = 4) and control cases (n = 5) using the NIS-Elements software program. We also measured the average cell size, percentage of cells with enlarged nuclei, and percentage of cells with recognizable nucleoli for each of three representative islets. We also assessed the interobserver diagnostic concordance of ANHH between five experienced and seven less-experienced pathologists.

**Results:**

There was no significant difference in the number, maximum diameter, or total area of islets between the two groups, even after correcting for these parameters per unit area. However, the number of islets with low circularity (< 0.71) per total area of the pancreatic parenchyma was significantly larger in ANHH specimens than in controls. We also found that the percentage of cells with recognizable nucleoli was significantly higher in the ANHH group than in the controls. There were no significant differences in the average cell size or the number of cells with enlarged nuclei between the groups. The correct diagnosis rate with the blind test was 47.5% ± 6.12% for experienced pathologists and 50.0% ± 8.63% for less-experienced pathologists, with no significant differences noted.

**Conclusions:**

Low circularity, which indicates an irregular islet shape, referred to as “irregular shape and occasional enlargement of islets” and “lobulated islet structure” in a previous report, is a useful marker for diagnosing ANHH. An increased percentage of recognizable nucleoli, corresponding to “macronucleoli in β-cells,“ has potential diagnostic value.

**Supplementary Information:**

The online version contains supplementary material available at 10.1186/s13000-023-01403-y.

## Introduction

Persistent hyperinsulinemic hypoglycemia (PHH) is caused by impaired control of insulin release from non-neoplastic and neoplastic β-cells of the pancreatic islets [1]. The primary cause of PHH in infants is nesidioblastosis, while that in adults is insulinoma [1]. Nesidioblastosis is histologically characterized by diffuse or focal hypertrophic β-cells in the pancreas, [2, 3] first described in infants [4]. Adult-onset nesidioblastosis was first reported in 1975, [5] and only a small number of adult-onset nesidioblastosis cases have since been described [1]. Adult-onset nesidioblastosis has recently been referred to as adult non-neoplastic hyperinsulinemic hypoglycemia (ANHH) [6] and is estimated to account for 0.5-5% of adult PHH cases [7].

The hypertrophic β-cells in ANHH are thought to be dysregulated in their function as a result of abnormal pathological features [8]. Anlauf et al. proposed diagnostic criteria for this disease, as shown in Table 1 [9]. Each of the major criteria as well as the minor criterion “macronucleoli in β-cells” seem to be objectively confirmed by a careful microscopic examination supported by insulin and Ki-67 immunohistochemistry. However, we believe that inter- and intraobserver variability can exist in the confirmation of the minor criteria “irregular shape and occasional enlargement of islets,” “increased number of islets,” and “lobulated islet structure,” owing to the lack of a quantitative standard for these items.

**Table 1:** Histopathologic criteria for the diagnosis of diffuse nesidioblastosis in adults (recently known as ANHH) proposed by Anlauf et al.

MAJOR CRITERIA	MINOR CRITERIA
Exclusion of an insulinoma by macroscopic, microscopic, and immunohistochemical examinations	Irregular shape and occasional enlargement of islets
Multiple β-cells with an enlarged and hyperchromatic nucleus and abundant clear cytoplasm	Increased number of islets
Islets with normal spatial distribution of the various cell types	Lobulated islet structure
No proliferative activity of endocrine cells	Macronucleoli in β-cells

Therefore, we established more quantitative criteria to evaluate the morphology of islets in ANHH specimens by comparing them with those in control specimens.

## Materials and methods

### Patients

We identified four cases of ANHH in the pathological database of Kyoto University Hospital between 1988 and 2022. The clinicopathological features of the patients are summarized in Table 2A. All four cases had PHH. Two of the four cases (Cases 1 and 3) were suspected of having ANHH owing to the lack of tumor-like nodules on computed tomography (CT), and the other two cases (Cases 2 and 4) were suspected of having an insulinoma or ANHH before distal pancreatectomy. Two cases of the ANHH specimens used for the evaluation were from the pancreatic tail (cases 1 and 2), and the other two were from the pancreatic body (cases 3 and 4).

**Table 2:** Clinicopathological features of the current cases

(A) ANHH cases.					
	CASE 1	CASE 2	CASE 3	CASE 4	
Age (years)/Gender	24/M	54/M	49/F	34/F	
Symptom	HH	Hypoglycemia with LOC	Hypoglycemia with headache and LOC after TG	HH with arrhythmia	
Clinical Diagnosis	ANHH	Insulinoma	ANHH	Insulinoma	
CT scan	No nodule	No nodule	No nodule	No nodule	
Surgical procedure	DP	DP	DP	DP	
Postoperative course	No hypoglycemic symptom	Occasional hypoglycemic symptom	Repeated hypoglycemic symptoms 12 years after operation, she died of ischemic heart disease	No data	
Type	Diffuse	Diffuse	Diffuse	Diffuse	
Site of the pancreas in a representative section	Tail	Tail	Body	Body	
(B) Control cases.					
	CASE 1	CASE 2	CASE 3	CASE 4	CASE 5
Age (years)/Gender	74/F	46/F	65/M	69/M	65/M
Diseases	pulmonary vascular injury, AAV, HLH, aspergillosis, pulmonary nocardiosis	Ruptured esophageal varices, PSC, UC, LC	Systemic metastasis of prostate cancer	ICH, HT	AP, peritonitis, ALS
Site of the pancreas in a representative section	Tail	Tail	Head	Body	Body

AAV, anti-neutrophil cytoplasmic antibody (ANCA) associated vasculitis; ALS, amyotrophic lateral sclerosis; ANHH, adult nonneoplastic hyperinsulinemic hypoglycemia; AP, aspiration pneumonia; DP, distal pancreatectomy; HH hyperinsulinemic hypoglycemia; HLH, hemophagocytic lymphohistiocytosis; HT, hypertension; ICH, intracerebral hemorrhaging; LC, liver cirrhosis; LOC, loss of consciousness; PSC, primary sclerosing cholangitis; TG, total gastrectomy; UC, ulcerative colitis

Controls were obtained from autopsied pathological specimens at the Department of Diagnostic Pathology, Kyoto University Hospital, and the Department of Pathology, Iwate Medical University. Suitable conditions for controls were as follows: (1) no pancreatic tumor (including neuroendocrine tumor); (2) no metastasis or invasion of the tumor into the pancreatic parenchyma or peripancreatic tissue (adipose tissue, blood, or lymphatic vessels); (3) no history of diabetes; (4) no chronic pancreatitis; (5) no intensive systemic treatment; and (6) good fixation and no advanced autolysis. One control specimen was obtained from the pancreatic tail (cases 1 and 2), two from the pancreatic head (case 3), and two from the pancreatic body (cases 4 and 5). Clinicopathological features of the control cases are summarized in Table 2B.

### Pathological diagnoses

**Fig. 1:**
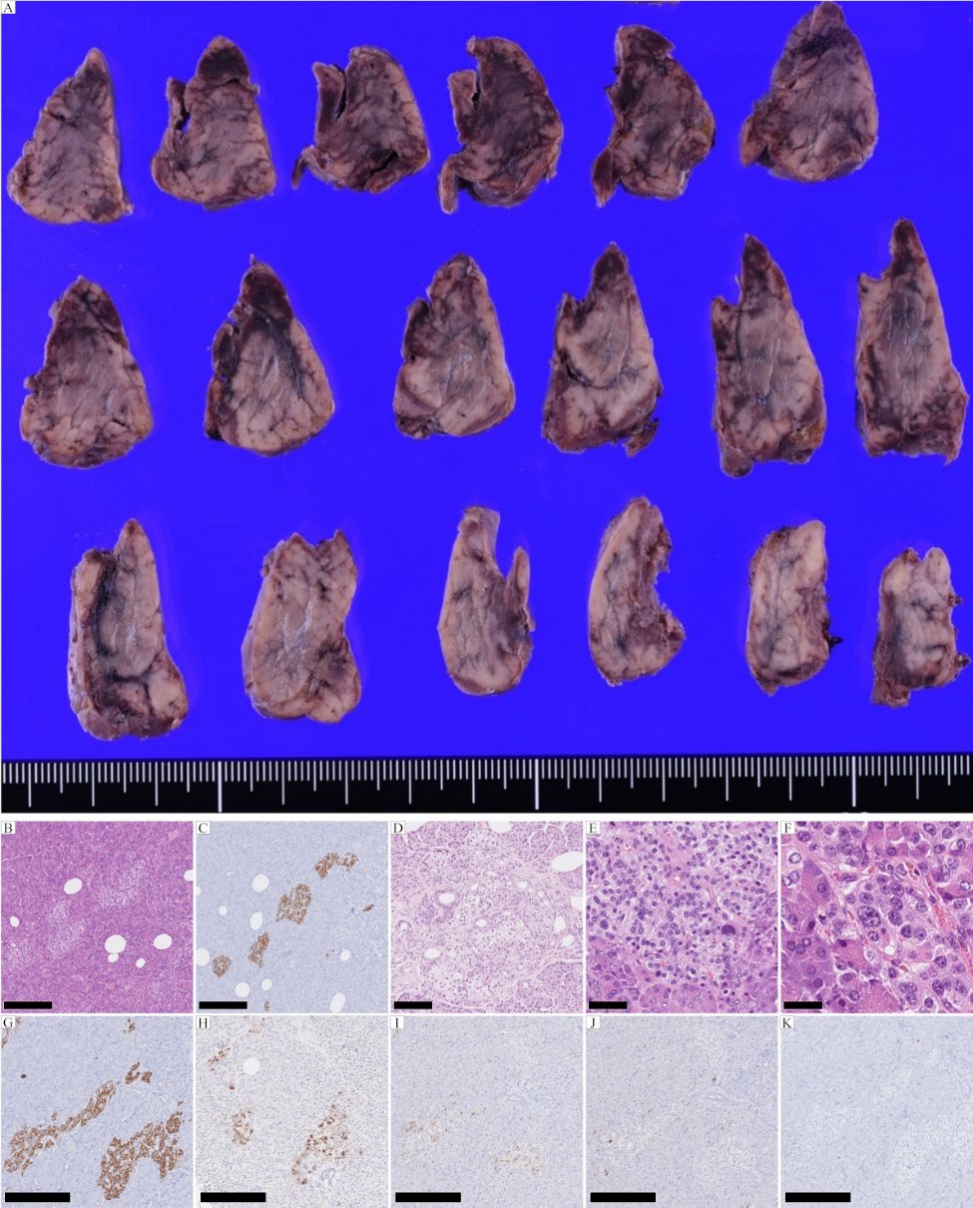
Pathological features of ANHH. **(A)** Macroscopic and **(B)** microscopic examinations (H&E) and **(C)** insulin immunostaining reveal no mass lesion in the sections of the resected pancreas. **(D)** Ductuloinsular complex. H&E. **(E)** Multiple β-cells with an enlarged and hyperchromatic nucleus and abundant clear cytoplasm. H&E. **(F)** Macronucleoli in β-cells. H&E. (G-J) Immunostaining. Islets with normal spatial distribution of the various cell types. **(G)** Insulin, **(H)** Glucagon, **(I)** Somatostatin, and **(J)** Pancreatic polypeptide staining. **(K)** Ki-67 immunostaining reveals no proliferative activity of the islet cells. Scale bars in B, C, G-K are 250 μm; the scale bar in D is 100 μm; the scale bar in E is 50 μm; and the scale bar in F is 25 μm

The pancreas in all four ANHH cases was entirely resected and examined (representative case shown in Fig. 1). All pancreatic specimens were cut perpendicular to the main pancreatic duct and preserved as formalin-fixed paraffin-embedded (FFPE) samples. Hematoxylin-eosin (H&E) staining and immunostaining for insulin (guinea pig polyclonal, Cat# A05664; DAKO, Glostrup, Denmark), glucagon (rabbit polyclonal, Cat# N1541; DAKO), pancreatic polypeptide (rabbit polyclonal, Cat# A0619; DAKO), somatostatin (rabbit polyclonal, Cat# N1551; DAKO), Ki-67 (mouse monoclonal, Cat# M7240; DAKO), and proliferating cell nuclear antigen (PCNA) (rabbit monoclonal, Cat# 13,110; Cell Signaling Technology, Boston, MA, USA) were performed using a Ventana Benchmark Ultra autoimmunostainer (Roche Diagnostics, Mannheim, Germany) according to the manufacturer’s protocols. H&E staining was also performed on all FFPE samples in the control cases, and immunostaining for insulin was performed in some cases. A microscopic examination was performed according to the criteria proposed in Ref. 8.

### The measurement of the numbers, maximum diameters, total areas, and circularities of the islets

**Fig. 2:**
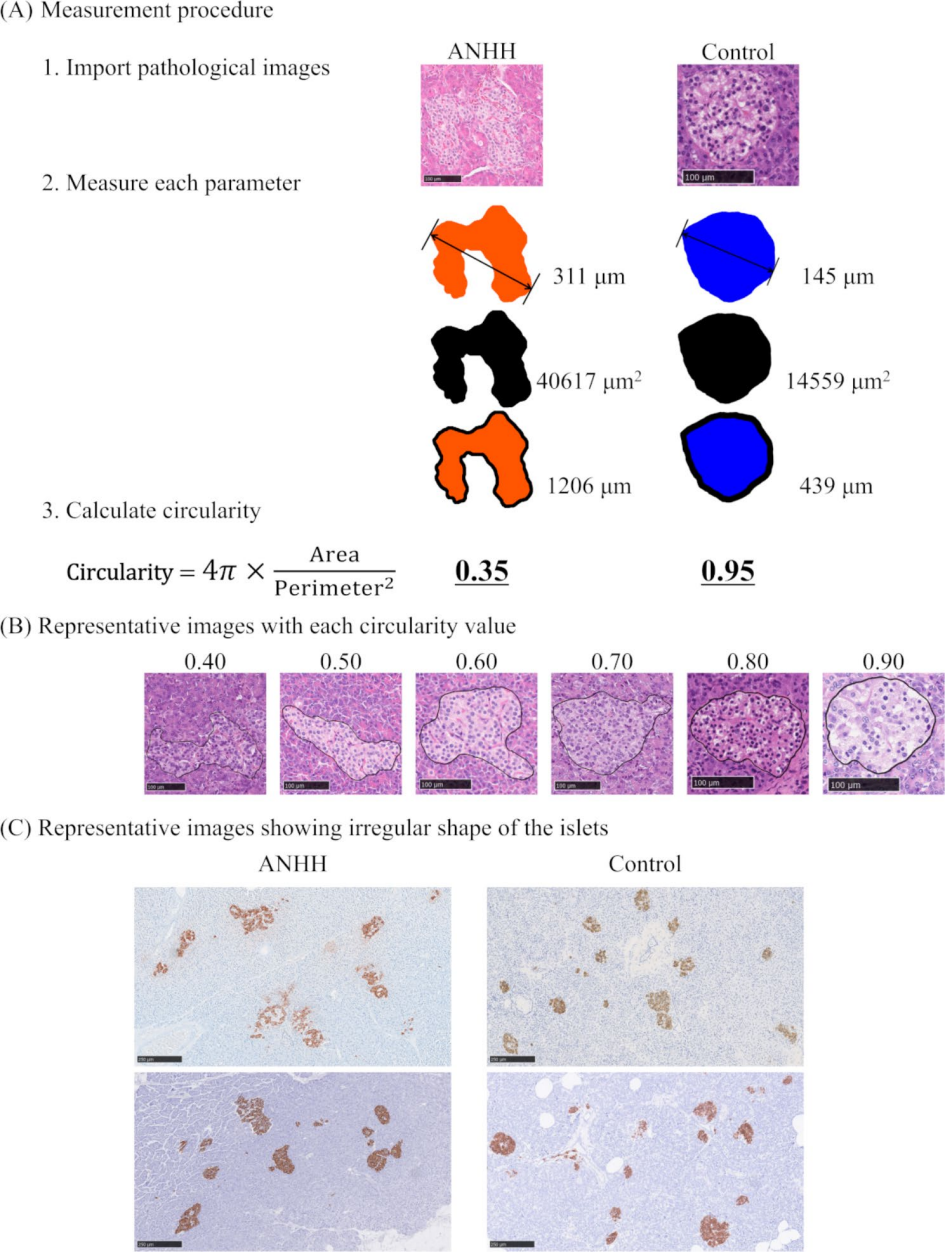
Measurement procedure of the islets **(A)**, representative images with each circularity value **(B)** and representative images showing an irregular shape of the islets in ANHH cases compared to controls (insulin immunostaining) **(C)**

Representative sections of ANHH and control specimens were digitized using whole-slide imaging (WSI) (Nanozoomer RS or Nanozoomer S360; Hamamatsu Photonics KK, Hamamatsu, Japan). The number, maximum diameter, perimeter, and area of each islet in each specimen were measured using an image analysis system (NIS-Element D software program, version 5.30.00; Nikon, Tokyo, Japan). Islets of any size were evaluated. The measurement method is shown in the schematic illustration (Fig. 2A). The cross-sectional area of pancreatic parenchyma was measured in the same manner. The circularity ranged from 0 to 1.0, with a higher circularity meaning that the shape of the given islet was closer to a circle [9–11]. In this study, circularity was calculated using the following formula: 4π × (area of islet) / (perimeter of islet)^2^. We adopted 0.71 as a threshold value because it was the value that generated the largest statistically significant difference.

### The measurement of cell size, the percentage of cells with enlarged nuclei, and the percentage of cells with recognizable nucleolus of the representative islets

For each of the three representative islets, we measured the average cell size (number of cells in the islet divided by the area of the islet), percentage of cells with enlarged nuclei (nucleus size: ≥9 μm), and percentage of cells in which the nucleoli could be recognized (nucleolus size: ≥1.5 μm) using the virtual slides above.

### Interobserver analyses

We conducted a blind assessment of the virtual slides of four ANHH cases (cases 1–4) and four control cases (cases 1–4) to evaluate interobserver diagnostic concordance. The evaluations were performed by 5 experienced pathologists (≥ 10 years’ experience) and 7 less-experienced pathologists (< 10 years’ experience). After presenting the diagnostic criteria in Table 1, pathologists were asked to identify whether they were ANHH or controls. The number of ANHH and controls are not presented.

### Statistical analyses

**Fig. 3:**
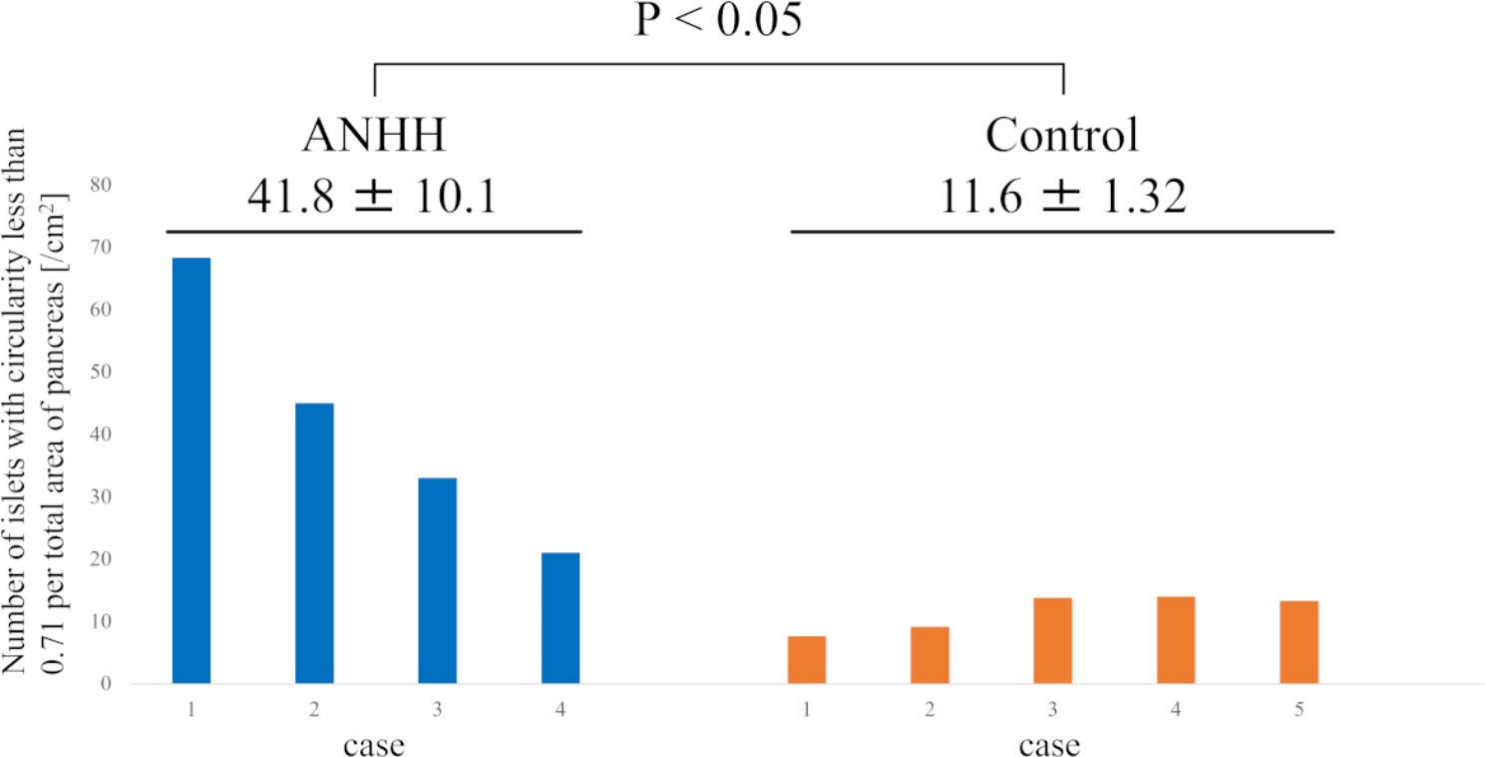
Number of islets with circularity < 0.71 per total area of pancreas parenchyma in patients with diffuse adult-onset ANHH compared with control specimens

Data in Table 3; Fig. 3 are expressed as the mean ± standard error. Differences between the groups were examined for statistical significance using an unpaired *t*-test (Excel; Microsoft, Redmond, WA, USA). *P* was set at p < 0.05.

**Table 3:** The measured parameters and aspect ratio of the islets of ANHH and control cases

	ANHH (n = 4)	Control (n = 5)	P value
Number of islets	417 ± 67.8	501 ± 90.0	0.50
Number of islets per total area of pancreas [/cm^2^]	211 ± 38.6	200 ± 25.4	0.80
Total area of islets [cm^2^]	0.0403 ± 0.0119	0.0474 ± 0.00916	0.64
Total area of islets per total area of pancreas	2.07% ± 0.661%	1.92% ± 0.315%	0.833
Maximum diameter [µm]	107 ± 14.0	105 ± 8.97	0.89
Number of islets with circularity < 0.71	82 ± 19.0	30 ± 5.71	0.023

## Results

### Macroscopic and microscopic examinations of the four ANHH cases

Macroscopically, all four cases lacked tumor-like nodules (representative case in Fig. 1). Immunohistochemically, proliferation markers, Ki-67 and PCNA were negative in islets of four ANNH cases (Fig. 1K and Supplementary Figure S1). Three of the four examined ANHH specimens met all major diagnostic criteria, and the remaining cases met three of the four major criteria (Table 4). Two cases met the minor criterion “macronucleoli in β-cells” (Table 4), and three cases showed “ductuloinsular complex”, which is non-specific but characteristic of ANHH (Table 4) [8]. The histopathological images are shown in Figs. 1 and 2.

**Table 4:** Concordance with each major and minor diagnostic criteria of ANHH in the current cases

Criteria	ANHH				Control				
Case	1	2	3	4	1	2	3	4	5
Exclusion of an insulinoma	P	P	P	P	P	P	P	P	P
Multiple β-cells with an enlarged nucleus (Diameter of nucleus is more than 15 μm)	P	N	P	P	N	P	P	P	P
Multiple β-cells with a hyperchromatic nucleus	P	P	P	P	N	P	P	P	N
Multiple β-cells with an abundant cytoplasm	P	P	P	P	P	P	P	P	P
Islets with normal spatial distribution of the various cell types	P	P	P	P	P	P	P	P	P
No proliferative activity of endocrine cells	P	P	P	P	P	P	P	P	P
Macronucleoli (Diameter of nucleolus is more than 5 μm)	P	N	P	N	N	P	P	N	N
Ductuloinsular complex	N	P	P	P	N	N	N	N	N

ANHH, adult nonneoplastic hyperinsulinemic hypoglycemia; N, negative; P, positive

### The measurement of the numbers, maximum diameters, total areas, and circularities of the islets in adult-onset ANHH

We measured and compared the number, maximum diameter, total area, and circularity of the islets in ANHH (n = 4) and control specimens (n = 5). No significant differences were noted in the number, maximum diameter, or total area of islets between the two groups (Table 3). We also compared these parameters when correcting per unit area of the pancreatic parenchyma, with no significant differences observed in the corrected parameters (Table 3). The average number of islets per specimen in the ANHH cases was 417 ± 67.8, and the average area was 199 ± 8.29 mm^2^; the average number of islets per specimen in the control cases was 501 ± 90.0, and the average area was 247 ± 27.7 mm^2^.

Next, we evaluated the circularity of the ANHH and control specimens, as in previous reports [9–11]. The number of islets with circularity < 0.71 per total area of the pancreatic parenchyma was significantly greater in the ANHH specimens than in the control specimens (Table 3; Fig. 3).

### The measurement of the cell size, percentage of cells with enlarged nuclei, and percentage of cells with recognizable nucleoli of the representative islets

We found that the percentage of nucleoli of islet cells with a recognized size in ANHH specimens was significantly higher than that in the control specimens (Supplementary Table S1). There was no significant difference in the average cell size or cells with enlarged nuclei of islet cells between ANHH and control cases.

### Interobserver analyses

The experienced pathologists correctly diagnosed ANHH in 47.5% ± 6.12% of cases, while the less-experienced did so in 50.0% ± 8.63% of cases, resulting in an overall correct diagnostic rate of 49.0% ± 5.43%, showing no significant differences (*p* = 0.83) (Supplementary Table S2).

## Discussion

ANHH is subclassified into focal and diffuse types, and almost all reported adult cases are of the diffuse type [2, 3, 8]. The current cases were all classified as the diffuse type. Therefore, our conclusion might be inappropriate for focal-type ANHH, which is difficult to differentiate from insulinoma [12, 13].

Anlauf et al. proposed the diagnostic criteria of adult-onset nesidiobalstosis (recently known as ANHH) [8]. All of the major criteria and the minor criterion “macronucleoli in β-cells” seemed to be objectively confirmed by a careful macroscopic examination and supported by insulin and Ki-67 immunohistochemistry. The percentage of recognizable nucleoli (nucleolus size: ≥1.5 μm) in representative islet cells of ANHH cases in the present study was significantly higher than that in the control cases. However, inter- and intraobserver variability may exist in efforts to confirm the minor criteria “irregular shape and occasional enlargement of islets,” “increased number of islets,” and “lobulated islet structure.” It is difficult to diagnose ANHH correctly, regardless of the pathologist’s experience. This may be due to a lack of prior clinical information and limited experience in diagnosing ANHH. Therefore, we explored quantitative assessment methods to help improve the diagnostic rate of ANHH.

The degree of circularity can reflect an “irregular shape and occasional enlargement of islets” and “lobulated islet structure” Thus, our finding that circularity is a marker for the diagnosis of ANHH seems acceptable. In contrast, an “increased number of islets” may not be useful for diagnosing ANHH. Circularity is calculated by the area and perimeter, which can be easily measured using an image analysis software program, such as NIS-Elements, ImageJ (https://imagej.nih.gov/ij/), or NDP viewer. However, if no such program is available, the images depicting different degrees of circularity in Fig. 2B C may help pathologists diagnose ANHH morphologically.

There was no significant difference in the percentage of cells with enlarged nuclei between ANHH and control specimens, but the percentage of islet cells with recognizable nucleoli was significantly higher in ANHH specimens than in control specimens. An increased percentage of islet cells with recognizable nucleoli may be a useful marker for diagnosing ANHH.

Extremely large islets, called “islet clusters”, have an area greater than 100,000 µm^2^ as defined in Ref. 14 and are sometimes found in the normal pancreas. In the current study, there was 1 islet with an area greater than 100,000 µm^2^ in the ANHH group and 5 in the control group. There was no marked difference in the islet size between ANHH and control specimens, and the average size of islets in the control cases was comparable to previously reported values [11, 14]. The presence of islet clusters might render “increased number of islets” and “occasional enlargement of islets” not useful for diagnosing ANHH.

## Conclusions

Circularity can be easily measured using digital slide images and an image analysis system. Low circularity (< 0.71) in islets should be considered to indicate an irregular islet shape and be taken as a useful marker for diagnosing ANHH, in contrast to the number, density, area, or maximum diameter of the islets, subsequent to the confirmation of PHH and the following major criteria: exclusion of insulinoma; multiple β-cells with enlarged and hyperchromatic nuclei and abundant, clear cytoplasm; islets with normal spatial distribution of various cell types; and no proliferative activity of endocrine cells. An increased percentage of recognizable nucleoli, corresponding to the minor criterion “macronucleoli in β-cells,“ thus has potential diagnostic value.

### Electronic supplementary material

Below is the link to the electronic supplementary material.

Supplementary Material 1: **Supplementary table S2** Interobserver Analyses.

Supplementary Material 2: **Supplementary table S1** The measured cell parameters for each of the three representative islets of the ANHH and control groups.

Supplementary Material 3: **Supplementary figure S1** (A) Islets of ANHH. H&E. (B) Proliferating cell nuclear antigen (PCNA) immunostaining reveals no proliferative activity of the islet cells. Scale bars = 250 μm.

## Data Availability

The dataset supporting the conclusions of this article is included within the article.

## Abbreviations

AAV: Anti-neutrophil cytoplasmic antibody (ANCA)-associated vasculitis
ALS: Amyotrophic lateral sclerosis
ANHH: Adult nonneoplastic hyperinsulinemic hypoglycemia
AP: Aspiration pneumonia
DP: Distal pancreatectomy
HH: Hyperinsulinemic hypoglycemia
HLH: Hemophagocytic lymphohistiocytosis
HT: Hypertension
ICH: Intracerebral hemorrhage
LC: Liver cirrhosis
LOC: Loss of consciousness
PCNA: Proliferating cell nuclear antigen
PSC: Primary sclerosing cholangitis
SE: Standard error
UC: Ulcerative colitis

## References

[1] Dravecka I, Lazurova I (2014). Nesidioblastosis in adults. Neoplasma.

[2] Heitz PU, Klöppel G, Häcki WH, Polak JM, Pearse AG (1977). Nesidioblastosis: the pathologic basis of persistent hyperinsulinemic hypoglycemia in infants. Morphologic and quantitative analysis of seven cases based on specific immunostaining and electron microscopy. Diabetes.

[3] Goossens A, Gepts W, Saudubray JM (1989). Diffuse and focal nesidioblastosis. A clinicopathological study of 24 patients with persistent neonatal hyperinsulinemic hypoglycemia. Am J Surg Pathol.

[4] Laidlaw GF (1938). Nesidioblastoma, the islet Tumor of the pancreas. Am J Pathol.

[5] Sandler R, Horwitz DL, Rubenstein AH, Kuzuya H (1975). Hypoglycemia and endogenous hyperinsulinism complicating Diabetes Mellitus. Application of the C-peptide assay to diagnosis and therapy. Am J Med.

[6] Sempoux C, Klöppel G (2023). Pathological features in non-neoplastic congenital and adult hyperinsulinism: from nesidioblastosis to current terminology and understanding. Endocr Relat Cancer.

[7] Yamada Y, Kitayama K, Oyachi M (2020). Nationwide survey of endogenous hyperinsulinemic hypoglycemia in Japan (2017–2018): congenital hyperinsulinism, insulinoma, non-insulinoma pancreatogenous hypoglycemia syndrome and insulin autoimmune syndrome (Hirata’s Disease). J Diabetes Investig.

[8] Anlauf M, Wieben D, Perren A (2005). Persistent hyperinsulinemic hypoglycemia in 15 adults with diffuse nesidioblastosis: diagnostic criteria, incidence, and characterization of beta-cell changes. Am J Surg Pathol.

[9] Kilimnik G, Jo J, Periwal V, Zielinski MC, Hara M (2012). Quantification of islet size and architecture. Islets.

[10] Olehnik SK, Fowler JL, Avramovich G, Hara M (2017). Quantitative analysis of intra- and inter-individual variability of human beta-cell mass. Sci Rep.

[11] Yu X, Zhang P, He Y (2021). A Smartphone-Fluidic Digital Imaging Analysis System for pancreatic islet Mass quantification. Front Bioeng Biotechnol.

[12] Kim JR, Jang JY, Shin YC (2016). Difficult diagnosis and localization of focal nesidioblastosis: clinical implications of (68)Gallium-DOTA-D-Phe(1)-Tyr(3)-octreotide PET scanning. Ann Surg Treat Res.

[13] Doi S, Yamada T, Kito Y (2021). Adult-onset focal nesidioblastosis with nodular formation mimicking Insulinoma. J Endocr Soc.

[14] Ionescu-Tirgoviste C, Gagniuc PA, Gubceac E (2015). A 3D map of the islet routes throughout the healthy human pancreas. Sci Rep.

