# Supplementary material for: Circularity of islets is a distinct marker for the pathological diagnosis of adult non-neoplastic hyperinsulinemic hypoglycemia using surgical specimens
Source: Diagn Pathol. 2023 Oct 20;18:115. doi: 10.1186/s13000-023-01403-y (PMC10588153; doi:10.1186/s13000-023-01403-y)
Supplement: Supplementary file 1 — Supplementary Material 1: Supplementary table S2 Interobserver Analyses. [file 13000_2023_1403_MOESM1_ESM.doc]

**Ref: Submission ID cfc7aa1d-d3ca-4e52-8ee3-0fc95e3927b3**

**Supplementary Table S2.** Interobserver Analyses.

| Randomized case number | 1 | 2 | 3 | 4 | 5 | 6 | 7 | 8 |
| --- | --- | --- | --- | --- | --- | --- | --- | --- |
| ANHH or control | A | C | A | C | C | A | A | C |
| Answers of pathologists |  |  |  |  |  |  |  |  |
| Experienced (years of experience) |  |  |  |  |  |  |  |  |
| #1 (30 yrs.) | A | C | A | C | C | A | A | C |
| #2 (16 yrs.) | A | C | C | A | C | A | C | C |
| #3 (16 yrs.) | A | C | A | A | C | A | C | A |
| #4 (12 yrs.) | C | A | C | A | C | A | A | A |
| #5 (11 yrs.) | C | C | C | A | A | A | C | C |
| Less-experienced (years of experience) |  |  |  |  |  |  |  |  |
| #6 (9 yrs.) | A | A | C | A | A | A | C | C |
| #7 (9 yrs.) | C | C | C | A | A | A | C | C |
| #8 (5 yrs.) | A | C | C | A | C | A | C | A |
| #9 (3 yrs.) | A | C | C | A | C | A | A | C |
| #10 (1 yrs.) | A | C | A | A | C | C | A | C |
| #11 (1 yrs.) | C | A | C | A | A | A | A | C |
| #12 (1 yrs.) | C | A | C | C | A | C | C | A |

A or ANHH, adult nonneoplastic hyperinsulinemic hypoglycemia; C, control.
