# Supplementary material for: Circularity of islets is a distinct marker for the pathological diagnosis of adult non-neoplastic hyperinsulinemic hypoglycemia using surgical specimens
Source: Diagn Pathol. 2023 Oct 20;18:115. doi: 10.1186/s13000-023-01403-y (PMC10588153; doi:10.1186/s13000-023-01403-y)
Supplement: Supplementary file 2 — Supplementary Material 2: Supplementary table S1 The measured cell parameters for each of the three representative islets of the ANHH and control groups. [file 13000_2023_1403_MOESM2_ESM.doc]

**Ref: Submission ID cfc7aa1d-d3ca-4e52-8ee3-0fc95e3927b3**

**Supplementary Table S1.** The measured cell parameters for each of the three representative islets of the ANHH and control groups.

|  | ANHH (n = 4) | Control (n = 5) | P value |
| --- | --- | --- | --- |
| Average area of representative islets [mm^2^] | 0.0411 ± 0.00689 | 0.0492 ± 0.00967 | 0.52 |
| Average number of representative islet cells | 272 ± 50.8 | 301 ± 49.8 | 0.67 |
| Average cell size [µm^2^] | 156 ± 7.65 | 159 ± 11.0 | 0.85 |
| Circularity of representative islets | 0.628 ± 0.0342 | 0.850 ± 0.0146 | <0.01 |
| Percentage of enlarged nuclei | 5.89% ± 1.42% | 3.62% ± 0.615% | 0.13 |
| Percentage of recognizable nucleoli | 9.38% ± 1.77% | 4.79% ± 0.956% | 0.024 |
