# Supplementary figures and images for: Circularity of islets is a distinct marker for the pathological diagnosis of adult non-neoplastic hyperinsulinemic hypoglycemia using surgical specimens
Source: Diagn Pathol. 2023 Oct 20;18:115. doi: 10.1186/s13000-023-01403-y (PMC10588153; doi:10.1186/s13000-023-01403-y)

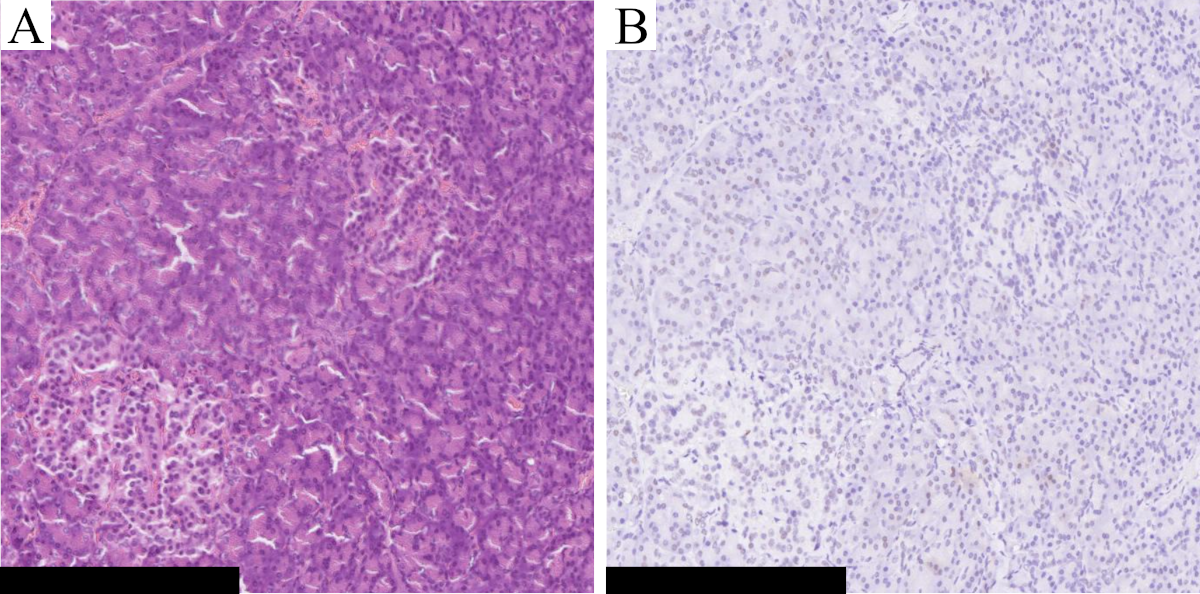

Supplement: Supplementary file 3 — Supplementary Material 3: Supplementary figure S1 (A) Islets of ANHH. H&E. (B) Proliferating cell nuclear antigen (PCNA) immunostaining reveals no proliferative activity of the islet cells. Scale bars = 250 μm. [file 13000_2023_1403_MOESM3_ESM.tif]
